# Alcohol consumption, endogenous estrogen and mammographic density among premenopausal women

**DOI:** 10.1186/s13058-015-0620-1

**Published:** 2015-08-07

**Authors:** Hanne Frydenberg, Vidar G. Flote, Ine M. Larsson, Emily S. Barrett, Anne-Sofie Furberg, Giske Ursin, Tom Wilsgaard, Peter T. Ellison, Anne McTiernan, Anette Hjartåker, Grazyna Jasienska, Inger Thune

**Affiliations:** The Cancer Centre, Oslo University Hospital, 0424 Oslo, Norway; Department of Obstetrics and Gynecology, University of Rochester School of Medicine and Dentistry, 601 Elmwood Avenue, Box 668, Rochester, NY 14534 USA; Department of Community Medicine, Faculty of Health Sciences, The Arctic University of Norway, 9037 Tromsø, Norway; Cancer Registry of Norway, PO Box 5313, Majorstuen, 0304 Oslo Norway; Department of Human Evolutionary Biology, Harvard University, Cambridge, MA 02138 USA; Fred Hutchinson Cancer Research Center, Seattle, WA 98109 USA; Department of Nutrition, Institute of Basic Medical Sciences, University of Oslo, 0316 Oslo, Norway; Department of Environmental Health, Jagiellonian University Collegium Medicum, 31-531 Krakow, Poland

## Abstract

**Introduction:**

Alcohol consumption may promote aromatization of androgens to estrogens, which may partly explain the observations linking alcohol consumption to higher breast cancer risk. Whether alcohol consumption is associated with endogenous estrogen levels, and mammographic density phenotypes in premenopausal women remains unclear.

**Methods:**

Alcohol consumption was collected by self-report and interview, using semi quantitative food frequency questionnaires, and a food diary during seven days of a menstrual cycle among 202 premenopausal women, participating in the Energy Balance and Breast Cancer Aspects (EBBA) study I. Estrogen was assessed in serum and daily in saliva across an entire menstrual cycle. Computer-assisted mammographic density (Madena) was obtained from digitized mammograms taken between days 7–12 of the menstrual cycle. Multivariable regression models were used to investigate the associations between alcohol consumption, endogenous estrogen and mammographic density phenotypes.

**Results:**

Current alcohol consumption was positively associated with endogenous estrogen, and absolute mammographic density. We observed 18 % higher mean salivary 17β-estradiol levels throughout the menstrual cycle, among women who consumed more than 10 g of alcohol per day compared to women who consumed less than 10 g of alcohol per day (*p* = 0.034). Long-term and past-year alcohol consumption was positively associated with mammographic density. We observed a positive association between alcohol consumption (past year) and absolute mammographic density; high alcohol consumers (≥7 drinks/week) had a mean absolute mammographic density of 46.17 cm^2^ (95 % confidence interval (CI) 39.39, 52.95), while low alcohol consumers (<1 drink/week) had a mean absolute mammographic density of 31.26 cm^2^ (95 % CI 25.89, 36.64) (p-trend 0.001). After adjustments, high consumers of alcohol (≥7 drinks/week), had 5.08 (95 % CI 1.82, 14.20) times higher odds of having absolute mammographic density above median (>32.4 cm^2^), compared to low (<1 drink/week) alcohol consumers.

**Conclusion:**

Alcohol consumption was positively associated with daily endogenous estrogen levels and mammographic density in premenopausal women. These associations could point to an important area of breast cancer prevention.

**Electronic supplementary material:**

The online version of this article (doi:10.1186/s13058-015-0620-1) contains supplementary material, which is available to authorized users.

## Introduction

High alcohol consumption has consistently been associated with breast cancer development [1–5], while a positive association between alcohol consumption and mammographic density, a strong independent biomarker for breast cancer development [6], has been observed in some studies [7–9], but not all [10]. In addition, few studies have included current and long-term alcohol consumption when studying the association between alcohol consumption and breast cancer risk [11]. Thus, whether there is an association between alcohol consumption and mammographic density phenotypes among premenopausal women remains unclear [12–14].

A positive association between endogenous estrogen and breast cancer development has consistently been observed [15, 16]. Interestingly, alcohol consumption may interfere with estrogen pathways, by influencing both aromatase activity, and therefore estrogen concentrations, and estrogen receptors [17–19]. Moreover, alcohol consumption may also interfere with the menstrual cycle by reducing variability and decreasing frequency of long cycles [20, 21]. Recently, high alcohol consumption was associated with endogenous luteal estrogen levels [22], and elevated weekend consumption of alcohol has been associated with higher peak levels of estrogens [3, 23]. Thus, one of the possible hypotheses suggested to explain the role of alcohol in breast cancer development is through its effect on the cumulative level of endogenous estrogen throughout life.

Importantly, assessment of endogenous sex steroid hormones among premenopausal women is complicated by intercyclic and intracyclic variations [24], but intercyclic variations seem to be less marked in nonseasonal and industrial populations [25]. Thus, given the substantial intracycle amplitude in hormone levels, at least 7–8 days/cycle should be measured but one cycle seems to be a valid measurement in regularly cycling women in a westernized society [24, 25]. Only a few studies have observed the association between endogenous estrogens levels and current and long-term alcohol consumption using both food diary and food frequency questionnaires (FFQ) in premenopausal women, and much remains unclear [22, 26]. Moreover, the association between alcohol consumption in combination with daily cyclic endogenous hormones and mammographic density phenotypes among premenopausal women remains unclear.

Recently, we observed a positive association between daily sex steroid hormones, growth factors, and mammographic density phenotypes [27, 28], while we previously have observed endogenous estrogen to be associated with important breast cancer risk factors including age at menarche and body composition [29–31]. These observations support the hypothesis that several lifestyle-related breast cancer risk factors might exert their effect on breast cancer development through sex steroid hormones.

On the basis of biological mechanisms suggested, and given that alcohol intake is a modifiable lifestyle factor and much remains unclear regarding the association between alcohol intake, endogenous estrogen, and mammographic density, there is a need for additional studies. The main aim of the present study was thus to examine the association between current, past-year, and long-term alcohol consumption, endogenous estrogen levels, and mammographic density phenotypes among premenopausal women.

## Methods

### Subjects and study design

A total of 204 women, aged 25–35 years, participated in the Norwegian Energy Balance and Breast Cancer Aspects I (EBBA-I) study during 2000–2002 at the Department of Clinical Research, University Hospital of North Norway (UNN), Tromsø [29]. Women were recruited from the general population by announcements in local newspapers and in public meeting places. Study subjects had to meet the following criteria, which were checked both in a telephone interview and in a personal interview by the same trained nurse during the entire study period: self-reported regular menstruation (cycle length: 22–38 days within the previous 3 months), no use of steroid contraceptives, pregnancy or lactation in the previous 6 months, no infertility, no history of gynecological disorders, and no chronic disorders (e.g. diabetes, hypothyroidism/hyperthyroidism) [29]. All women were fasting when attending three subsequent study visits throughout one menstrual cycle starting on the first day of menstrual bleeding: first visit, days 1–5 of the menstrual cycle (early follicular phase); second visit, days 7–12 (late follicular phase); and third visit, days 21–25 (late luteal phase). Two women were excluded owing to missing mammographic data, resulting in 202 women being included in the present study.

### Clinical examination and lifestyle factors

The participants underwent clinical examination at three scheduled visits by the same physicians. First attendance was on the first possible day after onset of menstrual bleeding at the Clinical Research Center, UNN, Tromsø, Norway. Height was measured to the nearest 0.5 cm, and weight to the nearest 0.1 kg on an electronic scale, body mass index (BMI) was calculated (in kg/m^2^), and waist circumference was measured [29]. Validated questionnaires (self-administered and interviewer administered) were used to collect information about reproductive history, previous hormone use, diet, and lifestyle factors [29, 31].

### Assessment of alcohol consumption

Alcohol consumption was assessed using a validated precoded food diary (Department of Nutrition, University of Oslo, Norway) and semi-quantitative FFQ. The food diary captured current alcohol consumption during seven selected days, representing each day of 1 week during the menstrual cycle (days 3–6 of the follicular phase and days 21–23 of the luteal phase) [32]. The semi-quantitative FFQ captured long-term alcohol consumption across the lifespan and alcohol consumption during the past year: “Have you ever drunk alcohol (yes/no)? If yes, how many glasses of wine, ½ liters of beer, fortified wine (0.4 dl) or measures of spirits did you drink on average per month at the following ages? (15–19 years, 20–24 years, 25–34 years).” “Are you currently an abstainer (yes/no)? If no, how many measures of alcohol have you consumed on average per month or per week in the past 12 months?” Both assessment methods were obtained by self-report, and were later checked for inconsistencies and missing values by trained interviewers.

### Assessment of estrogen

Overnight fasting serum concentrations of estrogen were assessed in fresh serum samples, taken at the three visits. The serum concentration of 17β-estradiol was measured using a direct immunometric assay (Immuno-1; Bayer Diagnostics UNN, Tromsø, Norway), and analyzed (Department of Clinical Chemistry, UNN, Tromsø, Norway). The sensitivity for estradiol was 0.01 nmol/l and the coefficient of variation (CV) was 3.9 %.

The participants collected daily morning saliva samples throughout one menstrual cycle, starting the first day of menstrual bleeding [33]. Collection of saliva in plastic tubes, pretreated with sodium azide, was done according to previously established validated protocols developed at the Reproductive Ecology Laboratory at Harvard University, Cambridge, MA, USA and the manufacturer’s protocol (Diagnostic Systems Laboratory, Webster, TX, USA) [33, 34]. The 17β-estradiol concentrations were measured in daily saliva samples using a ^125^I-based radioimmunoassay kit (#39100; Diagnostic Systems Laboratory, Webster, TX, USA). The samples were stored at −70 °C. All samples were run in duplicate, and samples from the same cycles were run within the same assay. The assays were carried out in different batches.

All cycles were aligned to the day of ovulation based on the identification of the drop in17β-estradiol, which provides a reasonable estimate of the day of ovulation [33, 35]. The mid-cycle 17β-estradiol drop could not be made for 14 of the participants: eight participants had too many missing days mid cycle, making it impossible to determine a drop day; and six participants had no discernible rise or drop in estradiol during the critical time window, and thus their cycles were unable to be aligned. These 14 women were therefore excluded from further analysis. The overall mean salivary 17β-estradiol concentration was calculated for all 204 women, whereas mid-menstrual 17β-estradiol (days −7 to +6) indices were calculated for the 190 women with aligned cycles. The sensitivity of the 17β-estradiol assay was 4 pmol/l, and average intra-assay variability was 9 %. The measurements of 17β-estradiol had higher CVs at the start and end of the menstrual cycle, and the interassay variability ranged from 23 % (low pool) to 13 % (high pool). Furthermore, there were higher rates of missing data at the end of the cycle, and thus we included 17β-estradiol salivary aligned measurements from day −7 to day +6 in this study.

### Assessment of mammographic density phenotypes

Bilateral two-view mammograms were obtained in the study from all women between cycle days 7 and 12 at the Centre of Breast Imaging, UNN, Tromsø, Norway using a standard protocol [36]. The left craniocaudal mammograms were digitized, and imported into a computerized mammographic density assessment program (Madena; University of Southern California School of Medicine, Los Angeles, CA, USA) [37]. One trained reader conducted the density measurements. The total breast area was defined using a special outlining tool, and the Madena software estimated size (in cm^2^) of this area. In order to assess density, the reader outlined a region of interest (ROI), and applied a tinting tool to pixels considered to represent dense areas of the mammograms within the ROI. The Madena software calculated the size of this dense area (in cm^2^). Absolute mammographic breast density represented this dense area, and percent mammographic density was the ratio of absolute mammographic breast density to total breast area. The mammograms were read in four batches, with an equal number of mammograms in each batch. A duplicate reading of 26 randomly selected mammograms from two of the batches showed an intraclass correlation coefficient for reliability of 0.94.

### Statistical analysis

Based on plausible suggested mechanisms linking alcohol to endogenous estrogen and to mammographic density, we studied the association between current and past alcohol intake alone, and in combination with endogenous estrogen levels and the study outcomes—percent and absolute mammographic density—using multivariate regression models. Previous observations in premenopausal [38] and postmenopausal [39] women have observed a twofold to threefold increase in breast cancer risk for women with percent mammographic density above 25 %. These observations support the comparison of women with above versus below median percent mammographic density. Mammographic density outcome variables were thus used as both continuous and dichotomized variables, representing lower and higher density using median values as cutoff points: percent mammographic density (28.5 %), and absolute mammographic density (32.4 cm^2^).

All variables, except alcohol consumption, were approximately normally distributed, allowing data analysis by parametric tests. Alcohol consumption was somewhat skewed and log transformation was performed prior to linear regression analysis; thus, the log transformation did not influence our results and the results are presented on the original scale. In addition, we log-transformed the hormone data owing to outliers; the results, however, were not influenced and the results are presented on the original scale.

Descriptive characteristics are presented as mean (standard deviation) or percent (number). Equality between categories of alcohol consumption was tested using one-way analysis of variance for continuous variables, and the chi-square test for binary variables. Based on suggested biological mechanisms influencing mammographic density, regression models including several potentially confounding variables were fitted. We tested whether adjustments for potentially confounding factors such as age (continuous), BMI (continuous), age at menarche (continuous), number of children (continuous), previous oral contraceptive (OR) use (categorical), current smoking habits (categorical), energy intake (continuous), and leisure-time activity (continuous) influenced our estimates. Age (continuous), BMI (continuous), number of children (continuous), current smoking habits (categorical), and previous OC use (categorical) were included as covariates in the final models. In regression models, alcohol consumption was considered both as a continuous variable and as a categorical variable, and the *p-*trend value was calculated based on continuous variables. Long-term alcohol consumption was defined as the average consumption from age 15 to present, and divided into categories: never/rarely, 1–4 drinks/month, and >1 drinks/week. Glasses of alcoholic beverages (past year and current) reported in both the FFQ and the food diary was converted into grams of alcohol by applying definitions of standard drinks for each beverage. Past year alcohol consumption was then divided into categories: total alcohol consumption (<1 drinks/week, 1–6 drinks/week, ≥7 drinks/week), and beer/wine/spirits consumption in past year (<1 drinks/week, ≥1 drink/week). Current alcohol consumption was defined as average consumption over 7 days and was divided in two categories: <1 drink/day and ≥1 drink/day. We defined one alcoholic drink as 10 g alcohol, which corresponds to about a small bottle (330 ml) of beer with 3.2 g alcohol per 100 ml, one glass (125 ml) of wine with 8.8 g alcohol per 100 ml, or a small glass (23 ml) of spirits with 31.7 g per 100 ml.

In the logistic regression models, alcohol consumption during the past year was used as a categorical variable, and the median split of mammographic density phenotypes was used as the dependent variable. One model was adjusted for age, and a multivariable model was further adjusted for BMI, number of children, current smoking habits, and previous OC use.

Multivariable adjusted, linear, mixed models for repeated measures were used to examine variation in daily salivary 17β-estradiol across the menstrual cycle, according to low (<1 drink/day) and high (≥1 drink/day) levels of current alcohol consumption. In addition, we stratified the analyses by median split of absolute mammographic density (32.4 cm^2^). The Toeplitz covariance structure was used in all models.

All statistical tests were two-sided using a significance level of *p* <0.05. Statistical analyses were conducted with SPSS version 21.0 (IBM Corporation, Armonk, NY, USA).

### Ethical consideration

All of the participating women signed an informed consent form, including taking a mammogram. The study protocol was reviewed and approved by the Regional Committee for Medical Research Ethics Northern Norway and the Norwegian Data Inspectorate (reference: 2001/3676 8 and 11).

## Results

Characteristics of the study population are presented in Table 1. Women who were high alcohol consumers (≥7 drinks/week, past year) tended to be younger, of older age at menarche, and had fewer children compared with low alcohol consumers (<1 drink/week, past year). Alcohol consumption in the past year was positively associated with both percent mammographic density and absolute mammographic density (Table 1). There was a moderate positive correlation between current and past-year alcohol consumption (Additional file 1).

**Table 1:** Characteristics of the study population by alcohol consumption (drinks/week) in the past year (FFQ), Norwegian EBBA-I study (*n* = 202)^a^

Variable	Overall (*n* = 202)^a^	Low consumers (<1 drink/week) (*n* = 66)^a^	Moderate consumers (1–6 drinks/week) (*n* = 96)^a^	High consumers (≥7 drinks/week) (*n* = 40)^a^	*p* value^b^
Characteristics					
Age (years)	30.7 (3.07)	31.17 (2.86)	30.75 (3.16)	29.87 (3.09)	0.105
Education (years)	16.1 (3.02)	15.82 (2.85)	15.98 (3.04)	16.70 (3.20)	0.324
Reproductive history					
Age at menarche (years)	13.1 (1.36)	12.75 (1.29)	13.21 (1.36)	13.48 (1.36)	0.016
Cycle length (days)	28.2 (3.17)	28.40 (4.13)	28.01 (2.78)	28.98 (3.5)	0.314
Parous (%)	51.2	61.2	46.0	27.5	0.002
Number of children (*n*)	0.91 (1.13)	1.30 (1.29)	0.80 (0.98)	0.53 (0.99)	0.001
Anthropometric measures^c^					
BMI (kg/m^2^)	24.4 (3.77)	24.47 (2.30)	24.55 (3.56)	23.92 (3.35)	0.665
Waist (cm)	79.5 (9.80)	76.69 (11.26)	80.28 (9.56)	77.44 (7.47)	0.300
Lifestyle factors					
Leisure time (MET/hour/week)	57.6 (88.6)	48.09 (33.44)	63.23 (121.18)	59.59 (45.16)	0.552
Oral contraceptive use					
Previous use (%)	82.7	76.1	85.0	90.0	0.119
Sum use (years)	3.7 (3.7)	3.27 (3.72)	3.99 (3.69)	3.84 (3.63)	0.458
Alcohol consumption in the past year					
Alcohol (drinks/week)	3.46 (4.10)	0.24 (0.28)	2.67 (1.46)	10.84 (2.87)	<0.001
Abstainers (%)	6.8				
Smoking habits					
Current smokers (%)	22.1	11.9	25.0	32.5	0.030
Former smokers (%)	45.4	32.8	50.0	55.0	0.037
Serum hormones^d^					
Estradiol (pmol/l)	146.7 (61.6)	145.5 (70.49)	147.4 (62.80)	146.8 (41.04)	0.983
Salivary hormones					
Estradiol, overall average^e^ (pmol/l)	17.9 (8.79)	17.68 (7.65)	17.61 (9.29)	18.98 (9.40)	0.691
Estradiol, mid-menstrual^f^ (pmol/l)	18.20 (8.98)	17.69 (7.52)	18.05 (9.69)	19.01 (9.59)	0.839
Mammograms^g^					
Percent mammographic density (%)	29.8 (19.0)	28.08 (19.05)	27.33 (18.02)	38.51 (19.30)	0.005
Absolute mammographic density (cm^2^)	34.7 (23.4)	31.57 (21.00)	31.89 (22.73)	46.77 (25.23)	0.001

Data presented as mean (standard deviation). One drink or unit is equal to 10 g alcohol

*BMI* body mass index, *EBBA-I* Norwegian Energy Balance and Breast Cancer Aspects I, *FFQ* food frequency questionnaire, *MET* metabolic equivalents

^a^Numbers may vary owing to missing information

^b^Equality between levels were tested using one-way analysis of variation for continuous variables and the chi-square test for binary variables

^c^Measurements at days 1–5 after onset of menstrual cycle

^d^Serum samples in early follicular phase: days 1–5 after onset of menstrual cycle

^e^Daily saliva samples throughout an entire menstrual cycle

^f^Daily saliva samples mid menstrual cycle, aligned cycle days −7 to 6

^g^Mammograms were taken days 7–12 (mid-cycle phase)

After adjustments, we observed an association between alcohol consumption in the past year and absolute mammographic density. High alcohol consumers (≥7 drinks/week) over the past year had a 47.6 % higher mean absolute mammographic density compared with low alcohol consumers (<1 drinks/week) over the past year (46.2 cm^2^ (95 % CI 39.4, 53.0) vs. 31.3 cm^2^ (95 % CI 25.9, 36.6) (*p*-trend 0.001)). The same positive association was observed with long-term alcohol use and absolute mammographic density (*p*-trend = 0.029) (Table 2).

**Table 2:** Multivariable adjusted means of mammographic density measures by alcohol consumption by long-term, past-year, and current consumption in premenopausal women (*n* = 202)^a^

Alcohol consumption^b^	Number	Percent density (%)	Absolute density (cm^2^)
Long-term, average^c^			
Never/rarely	7	32.14 (21.44, 42.84)	36.66 (20.17, 53.15)
1–4 drinks/month	134	26.74 (24.30, 29.17)	31.02 (27.27, 34.77)
>1 drinks/week	43	33.67 (29.27, 38.11)	42.08 (35.27, 48.89)
*p-*linear trend		0.051	0.029
Past year, average^d^			
<1 drinks/week	66	28.71 (25.23, 32.20)	31.26 (25.89, 36.64)
1–6 drinks/week	95	27.52 (24.73, 30.31)	32.17 (27.87, 36.48)
≥7 drinks/week	40	36.42 (32.02, 40.83)	46.17 (39.39, 52.95)
*p-*linear trend		0.003	0.001
Current, past week^e^			
<1 drink/day	143	28.11 (25.79, 30.42)	33.08 (29.46, 36.69)
≥1 drink/day	58	33.57 (29.87, 37.28)	40.38 (32.78, 44.35)
*p-*difference		0.016	0.121

Data presented as mean (95 % confidence interval). All analyses have used multivariate linear models

*FFQ* food frequency questionnaire

Adjusted for age (continuous), body mass index (continuous), number of children (continuous), previous oral contraceptives (categorical), and current smokers (categorical)

^a^Numbers may vary owing to missing information

^b^One drink or unit is equal to 10 g alcohol

^c^Alcohol consumption reported from age 15 to present time, FFQ

^d^Alcohol consumption reported in past year, FFQ

^e^Alcohol consumption reported in last 7 days representing 1 week, food diary

In multivariable linear regression analyses, we calculated estimated change in mammographic density per unit increase in the alcohol intake variable. Alcohol consumption in the past year was associated with both percent mammographic density (β-value 0.7, 95 % CI 0.2, 1.2) and absolute mammographic density (β-value 1.3, 95 % CI 0.5, 2.0). Similar associations were apparent, when past-year alcohol consumption was analyzed by alcohol type; for example, wine, beer, and others (Table 3). No association was observed between total current alcohol consumption and percent or absolute mammographic density (Table 3).

**Table 3:** Association between alcohol consumption by type of alcohol and mammographic density phenotypes in premenopausal women (*n* = 202)^a^ using multivariable linear regression models

Alcohol consumption	Percent density^b^ (%)	*p-*value	Absolute density^b^ (cm^2^)	*p-*value
Past year^c^				
Total alcohol (drinks/week^d^)	0.72 (0.23, 1.22)	0.004	1.28 (0.52, 2.03)	0.001
Beer (drinks/week^d^)	0.94 (0.15, 1.74)	0.021	1.87 (0.65, 3.09)	0.003
Wine (drinks/week^d^)	2.02 (0.62, 3.41)	0.005	2.84 (0.66, 5.01)	0.011
Others^e^ (drinks/week^d^)	1.74 (−0.23, 3.70)	0.084	3.17 (0.13, 6.21)	0.041
Current, past week^f^				
Total alcohol (g/day)	0.11 (−0.12, 0.34)	0.362	0.26 (−0.19, 0.61)	0.153
Beer (g/day)	1.71 (0.19, 3.22)	0.027	3.24 (0.91, 5.57)	0.007
Wine (g/day)	1.23 (−0.30, 2.75)	0.113	1.90 (−0.46, 4.25)	0.114
Others^e^ (g/day)	−0.15 (−1.93, 1.62)	0.865	−0.50 (−3.25, 2.26)	0.723

Data presented as β-value (95 % confidence interval). All analyses have used multivariable linear regression models, and are adjusted for age (continuous), body mass index (continuous), number of children (continuous), previous oral contraceptives (categorical), and current smokers (categorical)

*FFQ* food frequency questionnaire

^a^Numbers may vary owing to missing information

^b^Estimated change in mammographic density per unit increase in the alcohol intake variable

^c^Alcohol consumption reported in past year, FFQ

^d^One drink or unit is equal to 10 g alcohol

^e^Includes spirits and fortified wine

^f^Alcohol consumption reported in last 7 days representing 1 week, food diary

In age-adjusted logistic regression analyses, women with high alcohol consumption (≥7 drinks/week) had 2.4 (95 % CI 1.0, 5.6) and 4.4 (95 % CI 1.8, 10.9) higher odds of having high (above median) percent and absolute mammographic density, compared with women who were low (<1 drink/week) alcohol consumers. Similarly, in multivariable models we observed, among high consumers of alcohol, 5.1 (95 % CI 1.8, 14.2) times higher odds of having absolute mammographic density above median compared with low consumers. When we examined the type of alcohol consumed, high consumers (≥2 drinks/week) of wine had statistically significant 2.5 times higher odds ratio of having absolute mammographic density above the median, compared with low consumers (<2 drinks/week) of wine. However, our sample size was limited regarding the type of beverages (Table 4).

**Table 4:** Above-median percent (>28.5 %) and absolute (>32.4 cm^2^) mammographic density according to past-year alcohol consumption by type in premenopausal women (*n* = 202)^a^

Alcohol consumption, past year^b^		Age-adjusted model^c^		Multivariable-adjusted model^d^	
	Number	Percent density (%)	Absolute density (cm^2^)	Percent density (%)	Absolute density (cm^2^)
Total					
<1 drink/week	66	1.00	1.00	1.00	1.00
1–6 drinks/week	96	0.90 (0.47, 1.72)	1.10 (0.57, 2.11)	0.92 (0.39, 2.15)	1.16 (0.56, 2.42)
≥7 drinks/week	40	2.37 (1.00, 5.60)	4.38 (1.76, 10.87)	2.78 (0.90. 8.60)	5.08 (1.82, 14.20)
*p-*trend		0.094	0.004	0.106	0.004
Beer					
<2 drinks/week	156	1.00	1.00	1.00	1.00
≥2 drinks/week	45	1.33 (0.66, 2.67)	1.96 (0.97, 3.98)	1.25 (0.51, 3.03)	1.95 (0.90, 4.24)
*p-*difference		0.423	0.062	0.627	0.092
Wine					
<2 drinks/week	169	1.00	1.00	1.00	1.00
≥2 drinks/week	33	2.18 (0.97, 4.93)	2.60 (1.14, 5.93)	2.06 (0.77, 5.53)	2.47 (1.03, 5.92)
*p-*difference		0.060	0.024	0.152	0.043
Others^e^					
<2 drinks/week	184	1.00	1.00	1.00	1.00
≥2 drinks/week	18	2.80 (0.85, 9.17)	2.86 (0.88, 9.35)	2.55 (0.67, 9.64)	2.65 (0.79, 9.04)
*p-*difference		0.090	0.081	0.196	0.119

Data presented as odds ratio (95 % confidence interval). All analyses used multivariable logistic regression models

*FFQ* food frequency questionnaire

^a^Numbers may vary owing to missing information

^b^Alcohol consumption reported in past year, FFQ. One drink or unit is equal to 10 g alcohol

^c^Adjusted for age (continuous)

^d^Adjusted for age (continuous), body mass index (continuous), number of children (continuous), previous oral contraceptives (categorical), and current smokers (categorical)

^e^Includes spirits and fortified wine

**Fig. 1:**
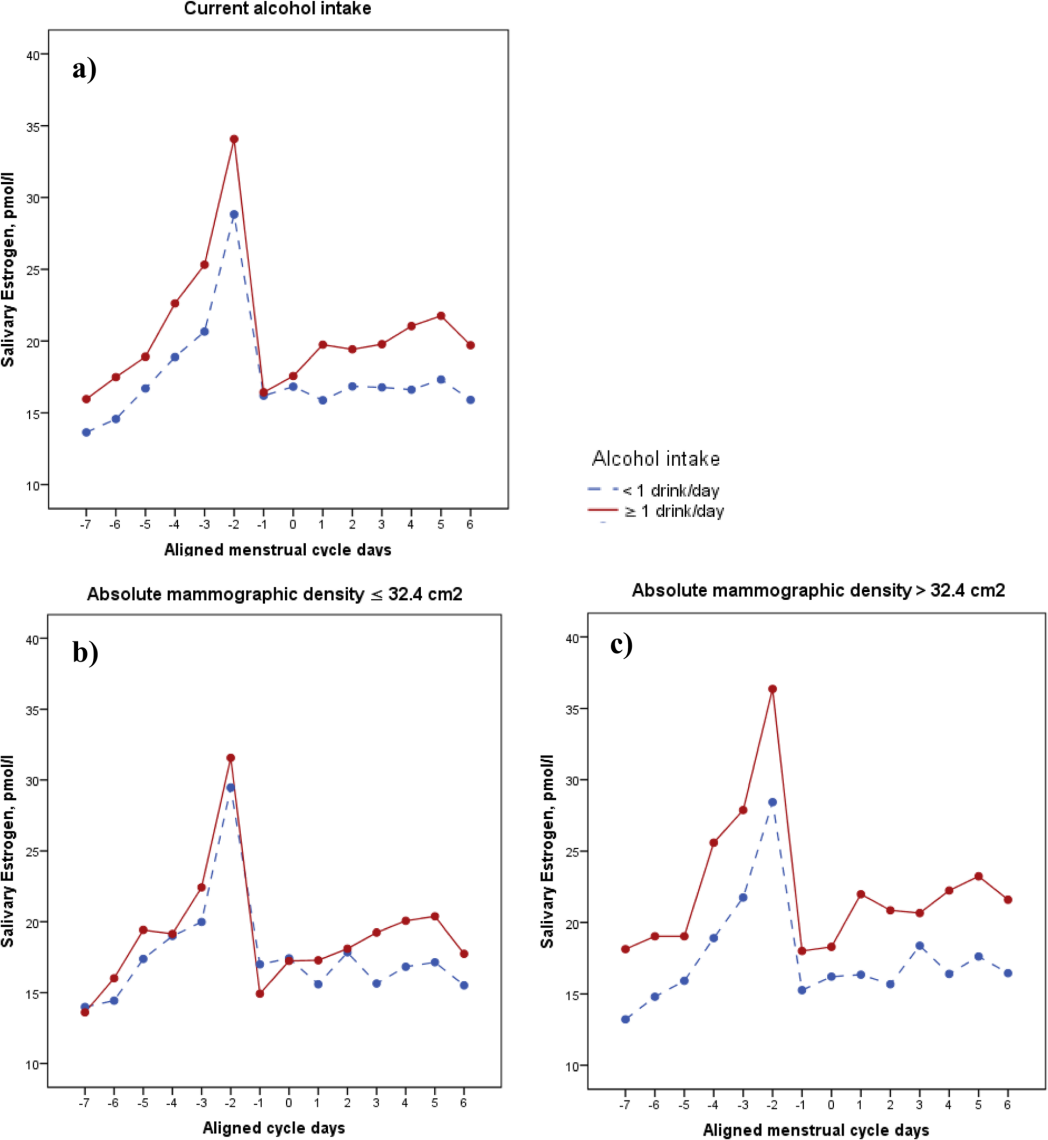
Adjusted mean salivary estrogen by high (*red line*) and low (*blue line*) current alcohol intake **a**–**c** stratified by absolute mammographic density **b**, **c** among 190 premenopausal women. **a** Current alcohol intake: <1 alcohol drink/day, 17.55 pmol/l; ≥1 alcohol drink/day, 20.70 pmol/l; *p* = 0.034. **b** Absolute mammographic density ≤2.4 cm^2^: <1 alcohol drink/day, 17.67 pmol/l; ≥ 1 alcohol drink/day, 19.09 pmol/l; *p* = 0.486. **c** Absolute mammographic density >32.4 cm^2^: ≤1 alcohol drink/day, 17.53 pmol/l; >1 alcohol drink/day, 22.36 pmol/l; *p =* 0.033*.* All analyses used linear mixed models for repeated measures, adjusted for age (continuous), body mass index (kg/m^2^, continuous), number of children (continuous), previous oral contraceptives (categorical), and current smokers (categorical). One drink or unit is equal to 10 g alcohol. Mean salivary estradiol throughout menstrual cycle

We found that women who consumed more than one unit of alcohol per day had 18 % higher mean salivary 17β-estradiol levels across the menstrual cycle compared with women who consumed less than one unit of alcohol per day (*p* = 0.034) (Fig. 1a). In analyses stratified by median absolute mammographic density, we observed that women with high current alcohol consumption (>1 drink/day) and high absolute mammographic density (>32.4 cm^2^) had 28 % higher mean salivary 17β-estradiol levels throughout the menstrual cycle, compared with women who consumed less than one unit of alcohol per day (*p* = 0.033) (Fig. 1c). No association was observed between current alcohol consumption and salivary 17β-estradiol in women with low absolute mammographic density (≤32.4 cm^2^) (Fig. 1b). Moreover, no associations were observed between alcohol consumption, salivary estrogen, and percent mammographic density. Of note, no associations were observed between alcohol consumption, serum estrogen, and mammographic density phenotypes (data not shown). To test for interaction between alcohol consumption and mammographic density, variables were first dichotomized and then entered into the linear mixed model with interaction terms. We observed no statistically significant interactions between alcohol consumption and mammographic density (data not shown).

## Discussion

In this study among premenopausal women, we observed positive associations between current alcohol consumption and endogenous estrogen levels, as women who reported consuming >10 g alcohol per day during the last week had 18 % higher mean 17β-estradiol level throughout the menstrual cycle compared with women who consumed <10 g alcohol per day during the last week. We also observed positive associations between past-year and long-term alcohol intake and percent and absolute mammographic density. Women who reported drinking ≥7 drinks/week over the past year had 48 % higher absolute mammographic density, compared with low alcohol consumers (<1 drinks/week). Similarly, high alcohol consumers in the past year had five times higher odds ratio of having above-median absolute mammographic density compared with low alcohol consumers during the past year. Of note, the current average alcohol consumption in our study was low, with a mean alcohol consumption of 2.9 drinks per week, and 18 % reported drinking more than one drink of alcohol per day (mean 6.7 g alcohol/day). Of note, the women reported drinking on average 11.3 g alcohol at weekends and on average 2.5 g alcohol per day on weekdays, which corresponds with levels of alcohol consumption observed in comparable populations [10]. Furthermore, we observed similar alcohol consumption by the two assessment methods used (semi-quantitative FFQ and food diary).

Owing to the limited number of participants included, and the limited variation in type of alcoholic beverage, we could not study whether a specific type of beverage influenced hormonal levels and mammographic density more than others. However, the confounders did not differ by type of beverage.

Our study is the first, to our knowledge, to study associations between current and long-term alcohol consumption and endogenous cyclic premenopausal estrogen levels and mammographic density phenotypes. Studies consistently support a modest association between alcohol consumption and breast cancer risk [1–5], with a suggestive dose–response relationship [1]. One of the possible hypotheses suggested to explain the role of alcohol in breast cancer development is through alcohol’s effect on the cumulative level of estrogen throughout life. However, inconsistent associations have been observed between alcohol and timed sampling of estrogens in the menstrual cycle [22]. Recently, a positive association between alcohol and luteal estrogen, but not between follicular estrogens, was observed [22]. These observations underline that additional studies are warranted to better understand the associations between alcohol and the cumulative level of estrogen throughout the menstrual cycle phase during premenopausal years. Alcohol consumption may act through increased aromatase activity [18], but other different biological mechanisms have been hypothesized [40, 41], supporting an association between endogenous estrogen and alcohol consumption. Interestingly, an association between endogenous estrogen and mammographic density has been observed [42–44]. The observed effect of high alcohol consumption on mammographic density may thus be explained by a direct effect of alcohol on breast density and/or an indirect effect through the estrogen pathway.

Previous studies report inconsistent results regarding the association between alcohol consumption during the past year and mammographic density phenotypes [7, 8, 10, 12, 45]. Few studies have examined the association between alcohol consumption and mammographic density in premenopausal women [12–14]. However, in some studies including both premenopausal and postmenopausal women, a positive association between alcohol consumption and qualitatively [7, 12] and quantitatively [13, 14] determined mammographic density was observed. Alcohol consumption over the past year reflects the cumulative alcohol consumption during 12 months, while current alcohol consumption assessed by our food diary reflects alcohol consumption during 1 week. The association observed in the present study between past-year alcohol consumption and mammographic density may thus be easier to observe compared with current alcohol consumption.

Interestingly, almost all of our observed associations were in relation to absolute mammographic density, also observed by others [45, 46]. Absolute mammographic density reflects dense areas of the breast, mainly composed of epithelial and stromal tissues, while percent mammographic density reflects relative amounts of fibroglandular and fat tissue [42, 47, 48]. Importantly, the absolute dense area is considered to represent the actual target tissue for tumor development, as ductal carcinoma in situ and invasive breast cancer more often occur in dense areas [49–51].

To our knowledge, our study—in contrast to others [46]—includes long-term, past-year, and current alcohol consumption by type of alcohol and total consumption during 7 days of one menstrual cycle, in relation to mammographic density phenotypes. Our study thus combines several unique features because alcohol consumption was assessed among premenopausal women both by self-report combined with a supplementary interview using validated semi-quantitative FFQ [52] as well as a food diary [32].

Estrogen was assessed in both serum and saliva throughout an entire menstrual cycle, following strict procedures and validated methods [33]. This is the recommended approach, yet it is rarely achieved owing to its logistic complexity [24]. However, serum estradiol includes both the protein-bound fraction and the free fraction of the hormone, while salivary estradiol reflects free, biologically active estradiol levels [29]. Of note, we observed no correlations between serum and salivary hormones among the participants in total, which is consistent with the results of other studies [53], as a correlation between salivary and serum concentrations within the individual was observed but not in total. This may be explained by the fact that serum hormones is dependent on the binding protein capacity, which differs greatly among individuals. Moreover, the serum levels in one individual, assessed only on a few days during a menstrual cycle, cannot be predicted from salivary concentrations in others [33, 53]. Exploratory non-invasive sampling of salivary hormones may thus provide novel insight into the associations between bioactive hormones, alcohol, and mammographic density in breast cancer research [53]. Of importance, the curves and salivary estrogen concentrations describing ovarian function in the presented EBBA-I study resemble those observed in other populations [35, 54].

Mammograms were obtained for study purposes justified by evaluation in ethical committees, during the late follicular phase (days 7–12 after first day of bleeding), avoiding possible variations in mammographic density during the menstrual cycle [55]. Thus, owing to safety concerns, we could only obtain one measure of mammographic density, and therefore could not measure density pattern changes over a menstrual cycle. The validated computer-assisted method used was read by one experienced blinded reader, and has previously been shown to give a superior prediction of breast cancer risk compared with qualitative methods [37, 56].

The present hypothesis-generating study also has some limitations. The small sample size limits further stratified analyses. However, our multiple salivary hormone variables are not considered to be independent measures, but indices within the same aligned menstrual cycle. Multiple corrections with Bonferroni for each variable would thus be too stringent. The assessment of daily salivary levels of unbound bioavailable estradiol throughout a menstrual cycle is unique, but there is a need for further studies because total serum hormones and free unbound salivary hormone levels are often correlated within individuals, while pooled data often show no significant correlations [33, 53]. Immunoassay methods used in the present study have most often been replaced recently by liquid chromatography–mass spectrometry, which compared with the immunoassay method is a more efficient way of analyzing salivary hormones with higher specificity and sensitivity. However, previous studies on estradiol measurements, specifically, have shown a high correlation between mass spectrometry and immunoassays [57]. The present study lasted for one menstrual cycle, and thus we were not able to capture any changes in estrogen levels between cycles. Alcohol consumption as assessed by a food diary was registered for 7 days (each day of a week represented), and captured binge versus daily consumption. The possibility to study daily variation for each cycle day was thus not possible. However, to minimize any information bias by day of the week or by cycle day, the current alcohol consumption was collected by a food diary for all seven unique days.

Given the increase in alcohol consumption among women worldwide [58], an association between alcohol consumption and breast cancer biomarkers, including endogenous estrogen levels and mammographic density, may have public health impact. If the association between alcohol use and mammographic density is determined to be causal, it could provide additional impetus for women to limit their alcohol consumption.

## Conclusion

Alcohol consumption was positively associated with mammographic density phenotypes in premenopausal women. Higher levels of daily estrogen were observed among women with high current alcohol consumption and high mammographic density. Given that alcohol intake consistently has been observed to be associated with higher breast cancer risk, these positive associations observed between alcohol consumption, endogenous estrogen, and mammographic density could point to an important area of breast cancer prevention. However, the results need to be replicated in larger studies, assessing information of both current and long-term alcohol consumption.

## Additional file

Additional file 1: Table S1. Presenting correlation between alcohol consumption assessed by FFQ and food diary (*n* = 202). (DOC 31 kb)

## Abbreviations

BMI: Body mass index
CI: Confidence interval
CV: Coefficient of variation
EBBA-I: Norwegian Energy Balance and Breast Cancer Aspects I
FFQ: Food frequency questionnaires
OC: Oral contraceptive
ROI: Region of interest
UNN: University Hospital of North Norway
