# Supplementary material for: Alcohol consumption, endogenous estrogen and mammographic density among premenopausal women
Source: Breast Cancer Res. 2015 Aug 7;17(1):103. doi: 10.1186/s13058-015-0620-1 (PMC4531831; doi:10.1186/s13058-015-0620-1)
Supplement: Additional file 1: Table S1. — Presenting correlation between alcohol consumption assessed by FFQ and food diary (n = 202). (DOC 31 kb) [file 13058_2015_620_MOESM1_ESM.doc]

**Additional file 1: Table S1:** Correlation between alcohol consumption assessed in FFQ and food diary (n=202)

|  |  | **Alcohol consumption** | |  |  |
| --- | --- | --- | --- | --- | --- |
| **Alcohol consumption** | Mean, g/day (SD) | Past year | Past week |  | *p-value* |
| Past year, FFQ | 4.95 (2.74) | 1^a^ | 0.57^a^ |  | *<0.001* |
| Past week, food diary | 6.72 (3.30) | 0.57^a^ | 1^a^ |  | *<0.001* |

Abbreviations: FFQ, food frequency questions; SD, standard deviation.

^a^Pearsons correlation coefficients
